# Reply to Villafuerte, R.; Funa, A. Comment on “Skjerve et al. Using Simulations to Help Public Health Students Overcome Language Barriers for Better Health Outcomes. *Int. J. Environ. Res. Public Health* 2023, *20*, 6259”

**DOI:** 10.3390/ijerph23060782

**Published:** 2026-06-11

**Authors:** Hilde Skjerve, Lars Erik Braaum, Ursula Småland Goth, Anette Sørensen

**Affiliations:** 1 School of Health Sciences, Kristiania University College, Kirkegata 24-26, 0152 Oslo, Norway; 2 Department of Intercultural Studies, NLA University College, Campus Oslo, P.B 7153 St. Olavs Plass, 0130 Oslo, Norway

We sincerely thank the authors of the comment [1] for the thoughtful and constructive comments on our article, Using Simulations to Help Public Health Students Overcome Language Barriers for Better Health Outcomes [2]. We appreciate that the comment recognizes both the relevance of the topic and the potential value of simulation-based learning in preparing public health students for communication across language barriers, and we are pleased that we can clarify the methodological intent and educational value.

## 1. Response to the Comment on the Methodical Intent

We agree that the study should be understood as an exploratory mixed-methods design. As stated in the original article, the study was based on facilitator logbooks, questionnaires, evaluation forms, and student reflections, and the interviews were not audio- or video-recorded. The letter points out that recordings of the simulated encounters would have strengthened the analysis by allowing closer examination of how communication strategies were enacted in practice. Several studies of simulation-based communication skills training have been conducted without video recording; instead, they place emphasis on the analysis of collected data and structured debriefing [3]. A literature review by Fernández-Alcántara (2024) shows that recent studies have developed and applied various virtual simulation tools for communication skills training among healthcare professionals, often using virtual patients and oral responses, which do not rely on video recordings [4]. To mitigate the attendant threats to credibility and transferability, we employed multiple strategies to strengthen analytic rigour. First, methodological triangulation, using facilitator logbooks, structured debriefing transcripts, participant questionnaires, and evaluation forms and the use of experienced role-players and facilitators to enhance scenario fidelity and consistency, was used to reduce bias. Second, a systematic, consensus-based coding of qualitative materials was conducted by multiple analysts to reduce individual bias, and the research team held ongoing reflexive discussions to surface and address potential observer effects. We reported these measures transparently, and, where feasible, future research can incorporate audio/video recording (with informed consent and secure data handling) or various virtual simulation tools, combined with conversation- or discourse-analytic methods and validated outcome measures, to permit more detailed analyses and stronger claims regarding transfer and generalizability.

## 2. Response to the Comment on Educational Value

De-Maria et al. found that cultural competence interventions remain limited in Europe [5]. Where implemented, these interventions have primarily sought to improve healthcare for minority populations, with an emphasis on individuals’ racial and ethnic backgrounds. Kreienbrink et al.’s scoping review of 16 publications identified 16 technological tools for addressing language barriers in healthcare. Usability was assessed in 13 studies [6]. The tools showed potential to support communication and diagnostic processes, but their use was limited by challenges related to translation accuracy, accessibility, and learnability. This indicates that technological solutions alone are insufficient to fully address language barriers in healthcare. Simulation-based training with clients experiencing language-related barriers is, therefore, necessary to ensure better health outcomes. Since publication, we have continued to develop this teaching approach. We have implemented the simulation-based teaching with two additional student cohorts. In one cohort, we did not introduce an additional teaching component but discussed the challenges of communicating with clients who speak little Norwegian or English. Based on this experience, we concluded that the topic needed to be addressed more systematically. In the most recent implementation, we therefore introduced dedicated lectures and seminars on communicating with clients who have limited skills in Norwegian or English. Subsequent teaching iterations reinforced the core conclusion of the original study: repeated simulation combined with structured debriefing reliably produces rapid, observable improvements in students’ capacity to manage language-discordant encounters. Across implementations, we consistently observed better performance in the second round of simulations, mirroring the original finding that debriefing fosters immediate learning and behavioural change. This aligns with the findings of Duff et al. (2024), who demonstrated that debriefing is a critical component of simulation experiences [7], as it enables reflection on, and discussion of, the concepts that emerged during the simulation [8].

These later teaching experiences support our original conclusion that students benefit from practising communication in situations where they cannot rely on a shared language. They also reinforce the value of debriefing, reflection, and repeated practice, which were central elements in the original simulation design. At the same time, we agree that future research should include robust methods to investigate whether learning gains are sustained over time and transferred to authentic public health settings.

In conclusion, we welcome the comments in the Letter to the Editor. They provide useful methodological reflections and point toward important directions for further research. Our subsequent teaching experiences have strengthened our view that simulation is a valuable pedagogical tool for preparing students to communicate with clients across language barriers, while also confirming the need for more systematic research on the long-term educational effects.

## 3. Concluding Remarks

We appreciate the Comment for drawing attention to the use of simulation in higher education for clients with language barriers. While acknowledging the inherent limitations of the methodology and long-term effects, we maintain that the scope and conclusions of our study remain valid.
